# Harmful Compounds and Willingness to Buy for Reduced-Additives Salami. An Outlook on Italian Consumers

**DOI:** 10.3390/ijerph16142605

**Published:** 2019-07-22

**Authors:** Giuseppe Di Vita, Simone Blanc, Teresina Mancuso, Stefano Massaglia, Giovanni La Via, Mario D’Amico

**Affiliations:** 1 Department of Agricultural, Forest and Food Sciences (DISAFA) University of Torino, Largo Paolo Braccini, 2, 10095 Grugliasco-Torino, Italy; 2 Department of Agriculture, Food and Environment (Di3A), University of Catania, Via S. Sofia 98-100, 95123 Catania, Italy

**Keywords:** conjoint analysis, consumer preference, processed meat product, consumer attitudes, low-salt content, nitrate-free

## Abstract

The consumption pattern of salami has been subjected to relatively widespread attention by academician, but few studies concerning the health implications of salami consumption have been published. Since additives and product origins represent important attributes for salami, the objective of this paper is twofold: (i) to explore the role of two additives, salt and nitrates, in addition to the Italian origin, in relation to consumer attitudes and preferences towards salami, (ii) to segment consumer behaviour by assessing their willingness-to-buy salami, in order to verify whether different purchasing patterns can be identified within the different social groups. The analysis was conducted on two different levels. The first was developed through a conjoint analysis, while the second implemented a frequency analysis based on a bivariate analysis. Results show the price as one of the most important variables in identifying the quality level of salami, in addition, certain socio-economic segments of consumers show a significant propensity to pay an additional price for salami with a low salt content and is nitrate-free.

## 1. Introduction

An increase in health issues linked to food consumption has been observed over recent years, and several authors have argued that consumers are conscious of the health risks associated with excessive meat consumption [1,2,3]. This change in consumer awareness has prompted processed meat companies to undertake various adaptive and innovative measures to satisfy the expectations of demand [4,5]. 

Such innovation has been directed towards the manufacture of productions that are more health-beneficial, and two different strategies have gradually been developed. The first strategy addresses the reduction of unhealthy compounds and ingredients, such as additives, fat, and salt [6,7]. The second is directed to enhance the nutritional contents of food, by adding compounds with health benefits, such as natural antioxidants like spices, omega 3 fatty acids, probiotics, etc. [8].

In the same context, another strand of literature highlighted the importance of the local or national origin of cured meat products. The geographical origin aspect of meat has been deeply analysed in terms of analytical determination [9], however, only a limited number of studies have argued the importance of this attribute for processed meat [10]. This topic has been addressed in a more specific way for fresh meat consumption. In this regard, one study, carried out on beef products, showed that a large number of consumers are willing to pay an additional premium for steak and hamburgers supplied with country-of-origin labelling [11]. Furthermore, another study confirmed that country-of-origin labelling strongly affects U.S. beef consumers, since they prefer domestic-originated beef, being prepared to pay an additional price for it [12]. In addition, the consumer likelihood of buying lamb meat was tested by Hersleth et al. [13], and the results enabled the authors to highlight the importance of geographic origin for Norwegian and Italian consumers. For processed meat products, a strong linkage between traditional dry fermented sausages and territory of origin was also found in a recent contribution [14], and specific attention has been paid to traditional cured meat products, such as salami [15].

The consumption pattern of salami has been subjected to relatively widespread attention by academician, and several studies have highlighted the role of information on consumer behaviour [16], while others have revealed how taste and local origin positively influence its consumption [17,18]. On the contrary, few studies concerning the health implications of salami consumption have been published. Among these, de Almeida et al. [18] showed that salami with a low salt content is less appreciated by consumers, whose preferences are mainly driven by taste. The same study observed the limited role of nutritional and health claims in salami consumers’ acceptance.

Since additives and product origins represent important attributes for cured meat products, the objective of this paper is twofold: firstly, to explore the role of two additives, salt and nitrates, in addition to the Italian origin, in relation to consumer attitudes and preferences towards cured meat products, by analysing the importance that these attributes have on the consumer perception of Italian salami. Secondly, to segment consumer behaviour by assessing their willingness-to-buy (WTB) of salami, in order to statistically verify whether different purchasing patterns can be identified within the different social groups. To strengthen our analysis, we assess WTB according to the socio-demographic characteristics of the sample, by correlating salami characteristics (with reduced salt content and nitrate-free) with gender, age, and sports activities.

## 2. Data Collection and Methodology

The study was carried out by developing both a qualitative and quantitative analysis over a representative sample of 499 Italian consumers. Data were collected by direct interviews of a random sample of processed meat consumers. Food shoppers were arbitrarily intercepted and interviewed at large retail stores during the act-of-buying process [19,20].

With this aim, we assessed the preferences for Italian salami consumption through a conjoint analysis (CA) and subsequently, a rank correlation between WTB and social and demographic characteristics of consumers was conducted.

CA represents a useful tool to verify whether consumers accept innovative products [21,22]. In this direction, several conjoint approaches have been adopted for novel and functional products [23,24]. Since it was also applied to a study on consumer behaviour of processed meat [25], and given that many studies have shown the negative effects of high salt and nitrite intake [26,27], we decided to employ this approach to evaluate consumer acceptance of salt-reduced and nitrate-absent salami, together with the role that national origin of salami has in the eye of consumers.

The attributes included in the CA were selected to evaluate consumer awareness towards health-beneficial attributes, in addition to the preference of origin of consumers, in highly differentiated salamis. Conjoint analysis was performed after identifying four different attributes, which were chosen according to recent contributions of current literature on processed meat consumer behaviour [28,29].

With this aim, we evaluated the perception consumers have towards salami with a potential reduction of salty taste and nitrite content, in conjunction with their Italian origin.

In order to categorise the attributes sequence and maximise the utility for the consumer, an additive linear composition was applied. Participants were shown eight different salami profiles, differing in terms of price per hectogram (1.30€; 1.90€; 3.00€), salt content (normal or reduced), nitrites (present/absent) and the importance of national origin (Italian/foreign). Subsequently, respondents were asked to select the attribute they would be most likely to buy according to their own level of acceptance [25]. The interviews were full-profile and were elaborated using SPSS 15.0 software (SPSS Inc., Chicago, IL, USA) for Windows. Table 1 shows the final subset of combinations of salami profiles.

In the second part of our study, we summarised the relationship between the most important variables analysed in the CA. Subsequently, a contingency table was prepared to summarise the relationship between the dependent variables, allowing the creation of a special type of frequency distribution table, where two variables are shown simultaneously [30].

Thus, we evaluated the WTB for salami with low salt content and nitrate-free, placing it in relation to the following independent variables: gender, age, sports activity. 

The dependent variable was measured at the ordinal level, while the independent variables consisted of categorical classes. Therefore, two rank-based non-parametric tests were used in order to verify whether significant differences between groups of independent variables and the ordinal dependent variable existed. The Mann-Whitney U test was used to compare differences between the two independent groups when the independent variable consisted of two categorical groups (gender, sports activity). Additionally, the Kruskal-Wallis H test was used in the case of the three categorical independent groups (age) [31].

## 3. Results 

### 3.1. Conjoint Analysis

CA allowed the assessment of the part-worth utility derived from the algorithm based on four different attributes and nine levels. The values of Pearson’s R and Kendall’s tau enable the verification that the conjoint analysis fits the data well.

The first results obtained with the conjoint analysis show the high relevance of variables linked to additives: nitrite-free and salt reduction in consumer choices. On the contrary, the importance of national origin attributes of salami has not been fully confirmed, as previously foreseen in the initial hypothesis.

Table 2 reports the main results of the conjoint analysis. By observing the part-worth utilities of attribute and their levels, the high importance that consumers pay to price emerges. In fact, price is considered as the most important variable in the quality detection of salami. Furthermore, a surprising apparent contradiction is that in spite of the cultural heritage one would assume that Italian salami represents in the eye of the consumer, for the sampled respondents, the origin is not perceived as an important attribute.

As already mentioned, respondents are not particularly attracted to the Italian origin of salami. This outcome evidences a low linkage between territory and product, and unlike the findings for other foodstuffs, where the origin only plays an important role as a quality marker, when it is associated with the safety or the higher quality of food [32], the variable linked to the national origin of salami has a limited relevance in consumer choice. Indeed, the latter is not only influenced by the price but also by other determinants, which combine sensory attributes (such as the lower saltiness) and health awareness aspects (absence of nitrates).

The ideal profile of salami is reported in Table 2. The best quality salami for respondents would be a product with the highest average price (3.00 €/hg), with a low salt content, without nitrites and, in a more nuanced way, with an Italian origin.

### 3.2. Frequency Analysis

Since high importance was revealed by the CA for the price and the two health variables, such as the absence of nitrates and the reduction of salt content, we decided to carry out a subsequent frequency analysis to detect potential differences among consumer behaviour, according to the socio-demographic characteristics of the sample. This was also performed to stress the role played by gender, age, and lifestyle, relating to the willingness-to-buy of consumers, and their willingness to pay an additional price for health-beneficial salami.

As reported in Table 3, our data shows that male WTB for salami with salt-reduced content was statistically significant and higher than the female WTB (*p* = 0.000). Additionally, physically active people demonstrate a willingness to pay higher than those who lead a sedentary life (*p* = 0.050). It emerges that 45% of women have no interest in paying an additional price for a product with less salt than they would pay for a traditional one, unlike the 65% of men who are willing to pay up to 20% more. Among those who practice sports activities and those who are sedentary, it emerges that as many as a third of them do not intend to pay more for a product with a low salt content. However, a small percentage (9%) of people practicing sports are willing to pay up to 30% more.

A Kruskal-Wallis H test showed that there was no statistically significant difference in the WTP behaviour score between the different age groups (*p* = 0.209).

As observed for the results on salami with reduced salt content, in relation to the nitrate-free salami (Table 4), there are no significant differences in the willingness to pay between subjects of different ages (*p* = 0.164).

In relation to the gender, significant differences emerge between the male and female groups (*p* = 0.000), but in this case, the behaviour is not uniform within each group. Compared to the willingness-to-buy products with a low salt content, we observe that consumers are, all in all, more vigilant towards products containing nitrates. In fact, 79% of females and 66% of males are willing to pay a premium price of +20% for salami with a low nitrate content. The same attitude emerges among those who lead a sedentary life (70% of them say it would correspond to a premium price of +20%) and among those who conduct physical activity (76%). Moreover, as in the previous case, physically active people are willing to pay more for products with a low nitrate content than sedentary ones (*p* = 0.001).

## 4. Discussion

The analysis was conducted on two different levels. The first was carried out through a CA analysis, while the second one was developed by the application of the frequency analysis. Results partially corroborate the initial hypotheses, in fact, respondents are willing to consume salami with a low content of additives, while the importance of national origin attribute of salami is not conceived as important by consumers.

The first result of the CA showed that the price is the most important attribute in the CA, thus corroborating the outcomes of recent surveys, showing that price strongly influences processed meat consumer choices [25]. As a consequence, this implies the low level of salami differentiation in the eye of consumers. In fact, unlike what has been observed for the reputation of highly differentiated products such as wine [33], the reputation of salami seems to be separate from the geographical origins.

Secondarily, consumers attach importance to the low salt content of salami, since the salami is perceived as a very salty product. As a consequence it would seem that consumers are aware of the importance of a low-sodium diet for their health [34]. This result confirms the findings of previous studies on salt reduction in food [21], given that consumers are willing to forego the salty taste of processed meats, as long as the texture and flavour remain unchanged [18,35].

The third most important attribute was the absence of nitrites. This outcome, which is entirely in line with previous studies [36], led us to detect a negative perception towards these additives, that are probably perceived as risky compounds for personal health. 

These overall outcomes confirm what has been reported in previous studies on processed meats, where a progressive reduction of nitrites and salt has observed for other processed meat products [29]. As a consequence, our results suggest the growing importance that consumers also ascribe to a reduction of additives in salami. In other words, as the quantity of additives is progressively reduced, the perception of the higher quality of the product increases. At the same time, these results are in line with the general trend of existing literature based on the innovation for healthier processed meat, which illustrates the need to reduce unhealthy substances as a strategy for the processed meat industry [8,37].

With reference to the scarcely relevant role played by origin, our results have implications for the existing literature on the origin of processed meats. In fact, despite traditional sausages and salami having a favourable reputation and obtaining a premium price based on quality certification [14,15], the reduction of harmful components is, in the eyes of the consumer, an element to be taken into higher consideration, deemed to be even more important than the geographic origin. In addition, salami would seem not to be perceived as a traditional Italian product, unlike what occurs in the case of dry-cured ham, which the consumer perceives as a more typical national product [38,39].

Concerning the frequency analysis, profound differences were found in the WTB for reduced-salt salami among genders, as men were more likely to pay an additional price, while women decisively were not. The results of our investigation were quite different when we considered the nitrate-free salami. In this case, both men and women, as well as physically active and sedentary respondents were prepared to pay a higher price. Consequently, it seems that salt is perceived as a harmful additive only by men and sports consumers, while nitrates are perceived as damaging compounds indistinctly by all the respondents. At the same time, we observed the irrelevant role played by age, which does not influence the willingness-to-buy a more health-beneficial salami. 

Finally, despite the fact that many studies revealed the strong influence of socio-economic factors, such as gender, age, and lifestyle [40,41,42,43,44] on traditional and healthy food product consumption, no relationship or comparison with the earlier studies can be developed in as much as, in the authors knowledge, the literature on socio-economic differences for more health-beneficial processed meat consumers is still relatively scarce. 

## 5. Conclusions

The paper explores, for the first time, the attitudes of consumers towards harmful compounds and willingness-to-buy reduced-additive salami, through a double statistic approach based on conjoint analysis and frequency analysis.

The findings of the conjoint analysis evidence, primarily, the role of price as the most important variable in identifying the quality level of processed meat products, since consumers show a high propensity to pay the highest prices for more health-beneficial salami, with a low salt content and nitrite-free. This outcome also highlights the high awareness of health issues by salami consumers and has an indirect implication also in terms of the sensory aspects of the ideal salami profile. A minor content of sodium implies, as a consequence, a reduction of the distinctive salty taste of the product, while the absence of nitrites has a direct effect on the colour, whereby the end result would be a less intense red colour. 

Concerning the frequency analysis, our findings confirm the strong statistical differences among the social groups, allowing the identification of the existence of a market segmentation according to the consumers’ willingness-to-buy. In particular, both genders and the respondents who play sports appear to be inclined to a reduction in the nitrate content of salami, being willing to pay an additional price for this. 

On the contrary, only men and sports-practicing respondents display a greater awareness of the risks deriving from the high salt content in processed meat and, as such, they demonstrate a greater propensity to purchase a salt-reduced salami.

A certain caution must be taken in interpreting these results. Although our analysis provided the description of the willingness to recognise a premium price for salami with a low salt content and being nitrate-free, and the identification of the differences between social groups, a limit of this research could lie in the qualitative methodological approach used. This approach provides the indication the two groups differ statistically from each other, however, it is not sufficient to verify the amount of values for each dependent variable. In other terms, the difference of values cannot be statistically quantified among specific groups. As a consequence, further findings are required to quantify the willingness to pay for salami with a healthier profile.

Notwithstanding the current efforts of the meat processing industry-led research that aims to mitigate the negative effects on human health of processed meat consumption by reducing additives, it is necessary to investigate the degree of awareness that the consumer currently has, and whether the consumer is willing to buy these processed meat products. 

Further studies are required in order to assess whether the low-salt content could affect the quality perception of salami consumers, while other strands of investigation should be addressed to analyse in depth the different purchasing patterns, including life-style and the awareness of health issues by processed meat consumers.

## Figures and Tables

**Table 1:** The profiles of eight salami profiles derived from orthogonal design.

Option	Price (€ hg^−1^)	Low-Salt Content	Presence of Nitrites	National Origin
1	1.90	Yes	No	Yes
2	1.30	Yes	Yes	No
3	3.00	Yes	Yes	Yes
4	1.90	No	Yes	No
5	3.00	No	No	No
6	1.30	No	No	Yes
7	3.00	No	Yes	Yes
8	3.00	Yes	No	No

**Table 2:** Conjoint analysis results.

Average Importance	Factor	Level	Utility
43.59%	Price (€/hg)	1.30	−0.7929
		1.90	0.2250
		3.00	0.5679
25.96%	Low-salt content	No	−0.4051
		Yes	0.4051
16.40%	Presence of nitrites	Yes	−0.2559
		No	0.2559
14.06%	Origin	Yes	0.2194
		No	−0.2194
Pearson’s R = 0.850			
Kendall’s tau = 0.500			

**Table 3:** WTB with reduced salt content.

Variable		“0”	+10%	+20%	+30%	+50%
		(%)	(%)	(%)	(%)	(%)
Gender	Male	24.7	17.5	47.5	8.7	1.5
	Female	44.9	24.8	22.6	6.0	1.7
	Mann-Whitney U	22,221				
	*p* value	0.000				
Age	18–29	33.7	18.7	37.4	9.6	0.5
	30–39	43.7	15.5	33.8	3.5	3.5
	≥40	26.8	28.0	35.7	8.3	1.2
	Kruskal-Wallis H	3.135				
	*p* value	0.209				
Sports	No	35.5	24.7	32.3	6.1	1.4
	Yes	32.6	16.1	40.4	9.2	1.8
	Mann-Whitney U	27,453				
	*p* value	0.050				

**Table 4:** WTB for nitrate-free salami.

Variable		“0”	+10%	+20%	+30%	+50%
		(%)	(%)	(%)	(%)	(%)
Gender	Male	22.4	43.0	23.2	9.1	2.3
	Female	17.0	23.4	55.7	2.1	1.7
	Mann-Whitney U	25,480				
	*p* value	0.000				
Age	18–29	17.1	33.2	38.5	10.2	1.1
	30–39	27.5	25.4	39.4	3.5	4.2
	≥40	16.6	41.4	37.9	3.0	1.2
	Kruskal-Wallis H	3.617				
	*p* value	0.164				
Sport	No	24.6	33.9	35.7	4.3	1.4
	Yes	13.8	33.5	42.2	7.8	2.8
	Mann-Whitney U	25,555				
	*p* value	0.001				
